# Construction of TERT Monoallelic Knockout and TERT Overexpression of Porcine Cell Lines and Study of the Cellular Biological Characteristics

**DOI:** 10.3390/ani16081227

**Published:** 2026-04-17

**Authors:** Yanhong Yang, Xiaojing Chen, Jing Wang, Jingjing Xiong, Xiaoyin Zhang, Jiaoxiang Wang, Weiwei Xu, Yubo Qing, Honghui Li, Hong-Ye Zhao

**Affiliations:** 1 Key Laboratory for Porcine Gene Editing and Xenotransplantation in Yunnan Province, Yunnan Agricultural University, Kunming 650201, China; 18314264541@163.com (Y.Y.); xiaojingchen1114@163.com (X.C.); kmwangjing@163.com (J.W.); bear111@foxmail.com (J.X.); 18788027723@163.com (X.Z.); jiaoxiangwang2021@126.com (J.W.); 18788562410@163.com (W.X.); qingyubo20@163.com (Y.Q.); honghui8300@aliyun.com (H.L.); 2 College of Veterinary Medicine, Yunnan Agricultural University, Kunming 650021, China; 3 College of Food Science and Technology, Yunnan Agricultural University, Kunming 650021, China

**Keywords:** TERT, monoallelic knockout, gene overexpression, porcine cell model, cellular biological characteristics

## Abstract

Telomerase reverse transcriptase subunit (TERT) is a key factor involved in telomere maintenance and genome stability, and the decline in its expression is closely related to cellular senescence. In this study, we established TERT monoallelic knockout (TERT+/−) and TERT overexpression (TERT-Over) cell lines in porcine iliac artery endothelial cells (PIEC) using CRISPR/Cas9 and PiggyBac systems to compare the effects of TERT monoallelic knockout versus overexpression on cellular biology. TERT expression and telomere length were assessed via qPCR and Western blot analysis. Cellular proliferation and senescence were evaluated using CCK-8 assays, cell cycle analysis, and SA-β-gal staining. Furthermore, the expression of key genes involved in cell proliferation, metabolism, and related signaling pathways was quantified using q-PCR. The results showed that the TERT mRNA level and telomere length decreased in TERT+/− cells. Meanwhile, we also observed that TERT+/− cells exhibited G1 phase arrest in the cell cycle, with suppressed proliferation and increased SA-β-gal-positive cells. This was accompanied by downregulation of cell cycle and proliferation-related genes, including c-Myc, the E2F family, and Ki-67, as well as downregulation of cell metabolism-related genes, including HIF1α, HK2, GLUT1, the SMAD family, FOXO1, and ATF4. In addition, cytochrome C was downregulated, suggesting activation of mitochondrial apoptotic signaling. Together, these findings indicate impaired proliferative and metabolic activity and are consistent with cellular senescence associated with telomere shortening. In TERT-overexpressing cells, the TERT gene expression and telomere length increase, cell proliferation accelerates, and the survival rate significantly increases under H_2_O_2_ treatment. This indicated that the overexpression of TERT can enhance resistance to oxidative stress, thus showing a kind of anti-aging phenotype. In conclusion, TERT monoallelic knockout induces cellular senescence-associated phenotypes in porcine endothelial cells, whereas TERT overexpression enhances proliferation and resistance to oxidative stress under the experimental conditions used in this study. The two porcine cell models established here may provide useful experimental materials for studying aging-related mechanisms and evaluating anti-aging interventions in large animals. Further studies are needed to directly determine their effects on cellular replicative lifespan.

## Simple Summary

Aging of cells is closely linked to a gradual loss of their ability to divide, function normally, and resist stress. Telomerase reverse transcriptase is an important molecule that helps maintain chromosome ends and protect genetic stability, and reduced levels of this molecule are often associated with cell aging. In this study, we created two pig endothelial cell models: one with reduced levels of telomerase reverse transcriptase and one with increased levels of telomerase reverse transcriptase. We found that cells with reduced levels of telomerase reverse transcriptase had shorter chromosome ends, grew more slowly, and showed more signs of senescence. In contrast, cells with increased telomerase reverse transcriptase had longer chromosome ends, grew faster, and were more resistant to oxidative stress, which is a harmful process linked to aging and tissue damage. These results show that lowering telomerase reverse transcriptase promotes cellular aging, while increasing it improves the cells’ anti-aging capacity. The pig cell models developed in this study provide useful tools for understanding how aging occurs and for testing possible anti-aging strategies in large animals, which may have value for both animal health and biomedical research.

## 1. Introduction

Telomeres play a crucial role in cellular differentiation, ageing, and disease [1]. The shortening of telomere length is recognized as a biomarker of ageing [2]. Telomere elongation is primarily mediated by telomerase activity (TA), a ribonucleoprotein complex composed of telomerase reverse transcriptase (TERT) and telomerase RNA component (TERC), which plays a key role in maintaining telomere length and genomic stability [3]. As the catalytic subunit of telomerase, TERT functions together with TERC to prevent telomere erosion, thereby averting replicative senescence and genetic instability. Furthermore, TERT exhibits additional non-telomeric activities, such as cell cycle regulation, modulation of cellular signalling and gene expression, prolongation of proliferative lifespan, and involvement in DNA damage responses [4]. Consequently, TERT is frequently employed as a target gene in constructing ageing models. TERT is typically highly expressed in actively dividing cells but suppressed in most somatic cells. When TERT expression declines, telomerase activity is impaired, limiting telomere repair capacity and leading to progressive telomere shortening. This triggers DNA damage responses and activates cell-cycle suppression pathways, such as p53/p21 and p16INK4a, causing cells to arrest in G1 and enter a senescent state [5]. Early seminal work by Bodnar et al. demonstrated for the first time that ectopic expression of hTERT in normal human diploid cells could extend cellular lifespan, thereby establishing a fundamental link between telomerase activity and replicative capacity [6]. Studies have shown that restoring TERT levels in aging animal models can reinitiate telomerase expression, alleviate tissue inflammation, and reverse aging phenotypes. This indicates that the inactivation of TERT and its multi-pathway cellular protective mechanisms is a key factor driving cellular aging and subsequent organismal aging [7]. The deletion of the TERT gene can accelerate telomere shortening and activate aging signals, enabling the stable and repeatable simulation of natural aging processes within a short timeframe. For instance, in systemic TERT knockout mice, telomeres progressively shorten from the first to the third generation, with the third generation exhibiting typical aging phenotypes accompanied by upregulation of p16INK4a and p21Cip1 [8]. In zebrafish, knocking out TERT can reduce its lifespan from approximately 42 months to 12–18 months, accompanied by significant degenerative changes in distal organs [9]. Studies have demonstrated that the deletion of TERT gene in mouse vascular endothelial cells can induce telomere-dependent DNA damage and cellular senescence, manifested as decreased capillary density, impaired vasodilation, mitochondrial dysfunction, and accelerated cardiovascular aging [10]. The knock-out of TERT gene can reproduce the telomere-dependent aging characteristics in multiple species and tissues. These studies provide a biologically relevant experimental model for investigating aging mechanisms and evaluating anti-aging strategies. However, there are few studies on the function of TERT gene in large animals, and further research on aging-related mechanisms in large animals is urgently needed.

CRISPR/Cas9 technology enables targeted gene disruption at specific genomic loci and is an important tool for constructing gene-edited animal models and studying gene function [11]. For example, Wang et al. reported the efficient generation of GHR-knockout Bama minipig fibroblast cells using CRISPR/Cas9, supporting the feasibility of CRISPR/Cas9-derived knockout models in pigs [12]. Consequently, gene knockout has become a vital tool for constructing high-fidelity disease models and elucidating pathological mechanisms. The PiggyBac transposon system recognizes TTAA sequences as recognition sites. It has both a relatively weak positional effect and a high vector capacity, and can integrate large fragments of exogenous DNA into safe genomic loci at a high frequency. Seamless excision is facilitated by transposase [13]. Therefore, it enables the efficient construction of gene overexpression models. Wu et al. compared the transpositional activity of four different transposons (SB11, Tol2, piggyBac, and Mos1) in four distinct mammalian cell lines and found that PiggyBac exhibited the highest transpositional activity [14]. Huang et al. compared the genomic integration efficiency and transpositional site preference of SB, Tol2, and PiggyBac transposons in peripheral blood lymphocytes (PBL) and umbilical cord blood (UCB)-derived primary T cells. The results showed that PiggyBac achieved the highest stable gene transfer efficiency in PBL and UCB T cells, surpassing SB11 and Tol2 [15]. Woltjen et al. utilized the PiggyBac transposon system to overexpress c-Myc, Klf4, Oct4, and Sox2, efficiently reprogramming human and mouse embryonic fibroblasts to obtain stable induced pluripotent stem cells (iPS). Subsequently, seamless excision of exogenous sequences by transposase confirmed that this method is more advantageous than traditional integrative viral methods for “clearing” reprogramming factors after functional verification [16]. PiggyBac has advantages over other overexpression strategies in terms of integration efficiency, reversibility, and adaptability to large payloads.

Compared with rodents, large animals, especially pigs, more closely resemble humans in physiological structure, organ function, metabolic characteristics, and lifespan. Therefore, pigs are considered a valuable large-animal model for ageing research. The rapid development of the aforementioned gene-editing technologies has made precise genetic manipulation in large animals increasingly feasible. Although TERT knockout or overexpression has been investigated in several previously reported models, studies on the parallel establishment of TERT monoallelic knockout and TERT-overexpressing porcine endothelial cell lines remain limited. In the present study, we generated TERT monoallelic knockout and TERT-overexpressing porcine iliac artery endothelial cell (PIEC) lines using gene-editing technology and performed a preliminary comparative analysis of their cellular biological characteristics. The novelty of this study lies in the construction of a paired porcine cell platform for comparative evaluation of TERT loss- and gain-of-function phenotypes in the same cellular background. This study not only provides new experimental materials for investigating TERT gene function, but also lays a technical foundation for the future generation of TERT-edited pig models using somatic cell nuclear transfer (SCNT).

## 2. Materials and Methods

### 2.1. Cell Culture

Porcine iliac artery endothelial cells (PIEC) used in this study were provided as a gift by the ShanghaiTech University (Shanghai Institute Cell Bank, Serial: GNO15, Shanghai, China). Cells were cultured in Dulbecco’s Modified Eagle Medium supplemented with 10% foetal bovine serum and 1% penicillin-streptomycin, and maintained at 37 °C in a humidified incubator with 5% CO_2_. Cell passage was performed using 0.25% Trypsin-EDTA solution (Yuanye, Shanghai, China). Cells were passaged or used for subsequent experiments when confluence reached 80–90%. This study was reviewed and approved by the Ethics Committee of Yunnan Agricultural University (approval No. YNAU202105009; publication date: 1 July 2021).

### 2.2. Construction of sgRNA Expression Vectors

Primers were designed for SNP detection within exons 2 and 3 of the TERT gene (Gene ID: 492280), respectively: TERT-SNP-F2: CTCTGCTGCGTCTCCCAG; TERT-SNP-R2: CCCAGTCTTTCAGGCTGTCA. PCR amplification was performed using genomic DNA from porcine iliac artery endothelial cells (PIECs) as the template. The amplified products were purified by agarose gel electrophoresis and subjected to Sanger sequencing to identify SNPs within the target regions. Based on the sequencing results, sgRNAs targeting the TERT gene were designed using the CRISPOR online platform (https://github.com/maximilianh/crisporWebsite, accessed on 12 March 2025), which was used for guide RNA design and target evaluation. The three highest-scoring sgRNAs for each exon region were selected for synthesis. The synthesized sgRNAs were diluted to 100 μmol/L and used as templates for PCR amplification. The amplified products were ligated into the pSpCas9(BB)-2A-GFP (px458) linearized plasmid, which had been digested with BsaI, and the ligation reaction was maintained at 16 °C for more than 2 h. The ligation products were then transformed into competent *E. coli* cells, plated on LB agar containing ampicillin, and incubated overnight at 37 °C. On the following day, a single colony was picked, bacterial lysate was prepared, and Sanger sequencing was performed for verification. Positive clones with correct sequencing results were selected for expansion, and plasmid DNA was extracted using a medium-scale endotoxin-free plasmid extraction kit for subsequent cell transfection experiments.

### 2.3. Construction of a PiggyBac Expression Vector for Overexpression of the TERT Gene

The porcine genomic sequence of the pEF-1α gene (Gene ID: FM995601) was downloaded from the NCBI database. Primers for amplification of the pEF-1α promoter were designed and synthesised (pEF-1αpromoter-F: CAAGGGCGGTGGAGAAGCCC; pEF-1αpromoter-R: TCACGACACCTAAGACGACA), and the promoter fragment was amplified by PCR using porcine genomic DNA as the template. In parallel, based on the cDNA sequence of porcine telomerase reverse transcriptase (pTERT; GenBank: NM_001244300.2), primers were designed and synthesised to amplify the pTERT coding sequence (pTERT-F: ATGCCGCGCGCGCCCCGGTG; pTERT-R: TCAGTCCAGGATGGTCCGGA). The pTERT fragment was amplified by PCR using porcine cDNA as the template. The amplified pEF-1α promoter fragment and pTERT fragment were digested with XbaI and MluI and ligated into the LD78-PB vector linearised with the same enzymes. The ligation reaction was performed at 16 °C for more than 2 h. The recombinant plasmid was then transformed into competent *E. coli* cells, plated on LB agar containing ampicillin, and incubated overnight at 37 °C. The following day, single colonies were screened by colony PCR and Sanger sequencing to identify positive clones with the correct insert sequence. Positive clones were expanded, and plasmid DNA was extracted using a medium-scale endotoxin-free plasmid extraction kit for subsequent cell transfection experiments.

### 2.4. Cell Transfection, Positive Clone Selection, and sgRNA Efficiency Assessment

For the TERT gene knockout plasmid (PX458 vector), the constructed sgRNA expression plasmid was mixed with the transfection reagent at a 1:1 ratio (10 μg:10 μg) and incubated before addition to PIECs. Twenty-four hours post-transfection, the medium was replaced with fresh complete medium containing penicillin (100 U/mL) and streptomycin (100 μg/mL). Fluorescent microscopy was performed at 48 h, followed by flow cytometric sorting of GFP-positive cells at 72 h. Following sorting, cells were centrifuged to remove supernatant, treated with cell lysis buffer, and lysed in a PCR machine using the following programme: 68 °C for 30 min, 16 °C for 5 min, 95 °C for 4 min, then maintained at 12 °C. Lysates were used for Sanger sequencing and T7E1 restriction enzyme digestion to assess the editing efficiency of each sgRNA. Following identification of the most efficient sgRNA, a second round of transfection was performed. Seventy-two hours post-transfection, positive cells were isolated via flow cytometric single-cell sorting to establish stable knockout cell lines.

For TERT overexpression experiments, after transfer to antibiotic-free medium, the pTERT-piggyBac plasmid and piggyBac transposase plasmid were mixed at a 1:2 ratio with the transfection reagent, incubated, and added to the cells. Twenty-four hours post-transfection, the medium was replaced with complete medium containing penicillin (100 U/mL) and streptomycin (100 μg/mL). After 72 h, puromycin was added at a final concentration of 2 μg/mL for drug selection. Following continuous selection for 3 days, surviving cells were harvested and expanded. Once the cells had stabilised, GFP and mCherry double-positive cells were sorted by flow cytometry and further subjected to cloning to obtain stable TERT-overexpressing cell lines.

### 2.5. Identification of Positive Clones

After culturing positive clonal cells for 12–14 days, the cells were harvested and centrifuged to remove supernatant. Cell lysis buffer was added, and samples were placed in a PCR machine for lysis. The lysis programme was set as follows: 68 °C for 30 min, 16 °C for 5 min, 95 °C for 4 min, followed by holding at 12 °C. The lysed DNA served as a template for verifying the TERT gene knockout.

Genotypic identification of TERT knockout cells was performed using three primer pairs for PCR amplification of the target region. Primer TERT-sg1-F: ACTGCTCTCTGCCCTTGTCTT; TERT-sg1-R: AGAGTGTGATGGGAAGGATAG amplified the sgRNA1 target sequence. Primer TERT-sg5-F: ACTGCTCTCTGCCCTTGTCTT; TERT-sg5-R: AGAGTGTGATGGGAAGGATAG amplified the sgRNA5 target fragment. Primers TERT-sg1-F and TERT-sg5-R amplify target fragments for sgRNA1 to sgRNA5. Following preliminary PCR validation, clonal sites exhibiting two characteristic amplification bands, indicative of large fragment deletion, were selected as candidate positive clones. Subsequently, the target region was subjected to further PCR amplification using three primer pairs. Purified PCR products were divided into two fractions: one for Sanger sequencing to validate mutation types, and the other for ligation into the 19T vector followed by transformation into competent cells. Positive clones were screened via bacterial culture PCR, and 8–10 positive colonies were selected for Sanger sequencing to definitively determine the TERT genotype in cells.

Following lysis of TERT-overexpressing cells, genomic DNA was extracted. Identification primers specific for the piggyBac vector, pTERT-p2a-egfp-F (CACCTGACACGAGCCAAAG) and pTERT-p2a-egfp-R (GGCGGTCACGAACTCCA), were used to amplify the target region. The presence of a specific amplification band indicated successful integration of the pTERT-piggyBac plasmid into the host cell genome, thereby confirming the clone as a positive TERT-overexpressing cell line.

### 2.6. qPCR

Following the collection of positive clonal cells, total cellular RNA was extracted using the TransZol Up kit (TaKaRa, Osaka, Japan). Reverse transcription was performed according to the PrimeScript RT Reagent Kit (TaKaRa, Osaka, Japan) protocol to convert the extracted RNA into cDNA. Subsequently, real-time quantitative PCR (qPCR) was performed using the TB Green Premix Ex Taq II (Tli RNaseH Plus) (TaKaRa, Osaka, Japan) kit according to the manufacturer’s protocol to detect gene transcription levels within the cells. GAPDH was selected as the internal reference gene for normalisation analysis.

### 2.7. Telomere Length Measurement in Cells

Following the extraction and quantification of cellular DNA, qPCR analysis was conducted to determine the average telomere length of each cell’s DNA using the TB Green Premix Ex Taq II (Tli RNaseH Plus) kit, adhering strictly to the manufacturer’s protocol. The primer sequences used for telomere length measurement were as follows: telomere primers, F: 5′-CGGTTTGTTTGGGTTTGGGTTTGGGTTTGGGTTTGGGTT-3′ and R: 5′-GGCTTGCCTTACCCTTACCCTTACCCTTACCCTTACCCT-3′; control gene (porcine 36B4 single-copy gene) primers, F: 5′-TGAAGTGCTTGACATCACCGAGGA-3′ and R: 5′-CTGCAGACATACGCTGGCAACATT-3′. The telomere PCR reaction system comprised 12.5 μL SYBR Green Mix, 400 nmol/L forward primer, and 400 nmol/L reverse primer. The 36B4 PCR reaction system comprised 12.5 μL SYBR Green Mix, 400 nmol/L forward primer, and 640 nmol/L reverse primer. Additionally, 35 ng of DNA was added to each well, with double-distilled water added to a final volume of 25 μL. Telomere and 36B4 reactions were conducted under identical conditions. Telomere relative length was expressed as the ratio of telomere signal to single-copy gene signal (T/S value).

### 2.8. Western Blot

Cells were lysed using RIPA lysis buffer (BOSTER, Wuhan, China) to extract cellular proteins. Protein concentration was determined using the BCA Protein Concentration Assay Kit (Beyotime, Shanghai, China), followed by incubation in a 100 °C water bath for 6 min. Equal amounts of protein (30 μg) were separated by 10% SDS-PAGE electrophoresis and transferred to a PVDF membrane. After blocking with 5% bovine serum albumin in PBST at room temperature for 2 h, the membrane was incubated overnight at 4 °C with the primary antibodies: anti-TERT antibody (BOSTER, Wuhan, China; 1:2000) and anti-β-actin antibody (Sigma, St. Louis, MO, USA; 1:5000). The following day, the membrane was washed three times with PBST for 10 min each and then incubated at room temperature for 2 h with the corresponding HRP-labelled secondary antibodies: Goat Anti-Rabbit IgG(H + L), HRP Conjugated (EpiZyme, Shanghai, China; 1:5000) for TERT, and Goat Anti-Mouse IgG(H + L), HRP Conjugated (EpiZyme, Shanghai, China; 1:10,000) for β-actin. Following membrane washing, signals were developed using an ECL chemiluminescent reagent (EpiZyme, Shanghai, China), and band images were captured using a Bio-Rad ChemiDoc MP imaging system. The grey values of the target protein bands were analysed using ImageJ 1.54 software and normalised to β-actin as an internal control to determine the relative protein expression levels.

### 2.9. Cell Cycle Detection

Cell cycle analysis was performed according to the instructions of the Cell Cycle Assay Kit (Beyotime, Shanghai, China). Adherent cells were first digested with trypsin, collected in medium, and centrifuged at 1000× *g* for 3 min. After removal of the supernatant, the cells were resuspended in 1 mL of pre-chilled PBS and centrifuged again. The supernatant was discarded, and the tube was gently tapped to prevent cell clumping. The cells were then fixed in 1 mL of pre-chilled 70% ethanol and kept at 4 °C for 30 min. After fixation, the cells were centrifuged at 1000× *g* for 3 min to remove the ethanol, resuspended in 1 mL of pre-chilled PBS, and washed once more under the same conditions. For staining, 0.5 mL staining buffer, 25 μL propidium iodide (PI) stock solution (20×), and 10 μL RNase A solution (50×) were mixed to prepare 0.535 mL of PI staining solution for each sample. Then, 0.5 mL of the prepared PI staining solution was added to each sample, and the cell pellet was gently resuspended. The samples were incubated at 37 °C in the dark for 30 min. Red fluorescence signals were subsequently detected using a flow cytometer (excitation wavelength: 488 nm), and forward scatter (FSC) and side scatter (SSC) were recorded simultaneously to reflect cell size and granularity. The acquired data were analysed to determine the DNA content distribution and the proportion of cells in each cell cycle phase (G0/G1, S, and G2/M).

### 2.10. CCK8 for Assaying Cell Proliferation

Using the Cell Counting Kit-8 (BOSTER, Wuhan, China), 100 μL of cell suspension was seeded into a 96-well plate, with approximately 2 × 10^3^ cells per well. Once cells had adhered and reached appropriate density, 10 μL of CCK-8 reagent was added to each well at designated time points (0 h, 24 h, 48 h). After gentle mixing, incubation continued for 1 h at 37 °C in a 5% CO_2_ incubator. Following incubation, the optical density (OD_450_) at 450 nm was measured using a microplate reader, with a blank well containing only medium and CCK-8 reagent but no cells serving as the background control.

### 2.11. Cellular Senescence Assay

SA-β-gal staining (Beyotime, Shanghai, China) was performed according to the manufacturer’s protocol. Cells were seeded into 6-well plates and cultured until they reached 80–90% confluence. The culture medium was then removed, and the cells were gently washed twice with PBS. Subsequently, 1 mL of SA-β-gal fixative was added to each well, and the cells were fixed at room temperature for 15 min. After fixation, the fixative was removed and the cells were washed twice with PBS. Then, 1 mL of staining solution was added to each well to ensure complete coverage of the cells. The plates were incubated at 37 °C in the dark for 12–16 h without CO_2_. After staining, the staining solution was discarded, and the cells were gently washed twice with PBS. Cellular morphology and staining patterns were then observed and photographed under an inverted microscope in different fields of view.

### 2.12. Clonogenic Assay

Cells in the logarithmic growth phase were seeded into 6-well plates at a density of approximately 500 cells per well, with three replicate wells for each group. The cells were cultured at 37 °C in a humidified incubator with 5% CO_2_ for 10–14 days until visible colonies had formed, with each colony containing approximately 50 or more cells. The culture medium was then discarded, and the cells were gently washed twice with pre-chilled PBS. The colonies were fixed with 4% paraformaldehyde (Servicebio, Wuhan, China) for 15 min, washed once with PBS, and stained with 0.1% crystal violet (Beyotime, Shanghai, China) solution at room temperature for 20 min. After staining, the dye solution was removed, and the plates were gently rinsed with clean water to reduce background staining, followed by air-drying at room temperature. Once completely dry, the colonies were photographed and recorded.

### 2.13. H_2_O_2_ Treatment and CCK-8 Assay

WT and TERT-over PIECs were seeded into 96-well plates at a density of 2 × 10^3^ cells/well. After cell attachment, the cells were treated with complete DMEM containing 200 μmol/L H_2_O_2_ for 0, 3, 6, and 9 h. The H_2_O_2_-containing medium (Beyotime, Shanghai, China) was prepared by adding 2.3 μL of 3% H_2_O_2_ stock solution to 10 mL DMEM supplemented with 10% FBS, followed by gentle mixing to obtain a final concentration of 200 μmol/L. Nine replicate wells were used for each time point. After treatment, the medium was replaced, and CCK-8 reagent (BOSTER, Wuhan, China) was added to assess cell viability according to the manufacturer’s instructions.

### 2.14. Statistical Analysis

Flow cytometry data were processed and analyzed using FlowJo_v10.8.1 software. All data were plotted and statistically analyzed using GraphPad Prism 10.4.0 software. Data are presented as mean ± standard deviation (mean ± SD). Statistical differences between two groups were analyzed using a two-tailed Student’s *t*-test, whereas comparisons among more than two groups were performed using one-way ANOVA followed by Tukey’s multiple comparisons test. The sample size (n) for each group is indicated in the corresponding figure legends. All experiments with statistical analysis and error bars represent independent biological experiments. A value of *p* < 0.05 was considered statistically significant. *p* < 0.05 (*) and *p* < 0.01 (**) indicate significant and highly significant differences compared with WT, respectively, while *p* < 0.05 (#) and *p* < 0.01 (##) indicate significant and highly significant differences compared with TERT-over, respectively.

## 3. Results

### 3.1. Establishment of TERT Monoallelic Knockout PIEC Lines

#### 3.1.1. SNP Detection at TERT Single-Allele Knockout Sites

**Figure 1:**
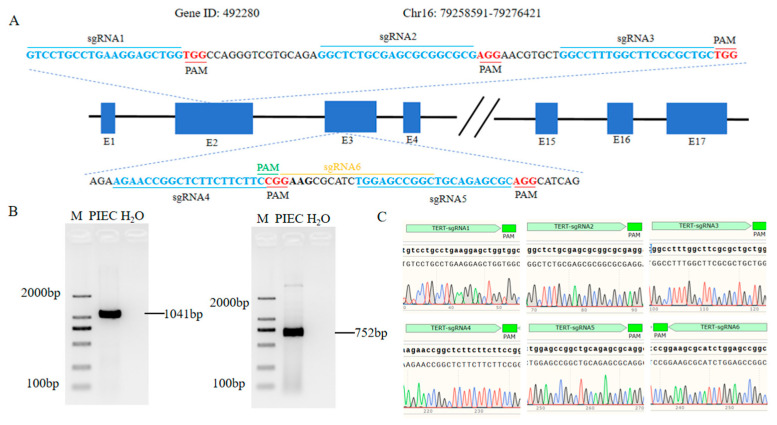
sgRNA target design and SNP validation in the target region. (**A**) Schematic diagram of sgRNA target design in exons 2 and 3 of the TERT gene. (**B**) Agarose gel electrophoresis results of PCR amplification products from the target region. (**C**) Sanger sequencing peak diagram of the target region.

To achieve precise editing at the target site and avoid reduced cleavage efficiency or increased off-target risk caused by sequence variation, SNP (single-nucleotide polymorphism) detection was first performed in the PIEC line. Based on the TERT gene sequence (Gene ID: 492280) on pig chromosome 16 in the NCBI database, six sgRNAs were designed to target exons 2 and 3 of the TERT gene (Figure 1A). PCR primers flanking these target regions were designed accordingly. Amplification using PIEC genomic DNA yielded specific fragments of 1041 bp and 752 bp (Figure 1B). Sanger sequencing showed clean chromatogram peaks without extraneous signals, and the obtained sequences matched the reference genome sequence exactly. These results indicated that no SNPs were present at the selected target sites in PIECs (Figure 1C), supporting their suitability for subsequent gene-editing experiments.

#### 3.1.2. Construction of TERT Single-Allele Knockout Vectors

**Figure 2:**
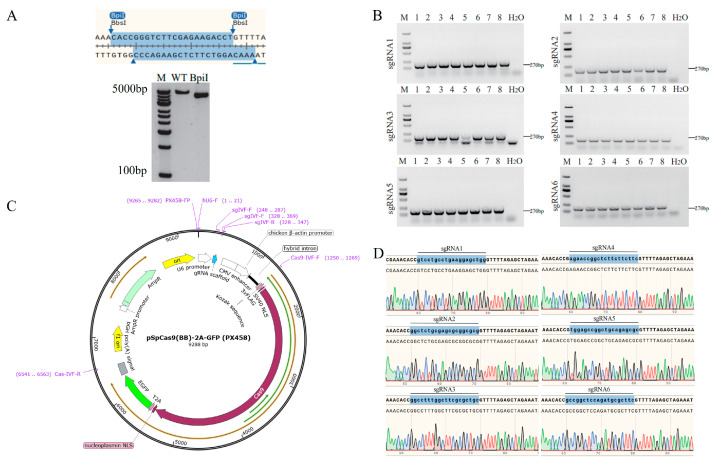
Construction of the sgRNA expression vector. (**A**) Schematic representation of the linearisation of the px458 plasmid following digestion with BpiI, along with the corresponding agarose gel electrophoresis results. (**B**) Electrophoresis patterns of PCR products from different sgRNA cloning colonies. (**C**) Schematic diagram of the px458 vector structure and sgRNA insertion site location. Arrows indicate the orientation or position of the corresponding genetic elements, different colors represent different functional regions of the vector, and the ellipsis indicates omitted non-essential vector sequences for schematic presentation only. (**D**) Sanger sequencing peak diagram of the sgRNA insertion region.

After confirming the absence of SNPs in the target sequence, the corresponding sgRNA oligonucleotide single strands were synthesised. Following annealing to form double strands, these were ligated with the px458 plasmid vector linearised by BpiI restriction enzyme (Figure 2A,C). The resulting ligation products were transformed into DH5α competent cells for cultivation. Eight random monoclonal colonies from each sgRNA ligation system were selected for PCR validation. Results demonstrated that all eight colonies amplified a single band (270 bp), consistent with the expected fragment size (Figure 2B). Subsequently, 1–2 positive clones from each sgRNA were validated via Sanger sequencing. Results confirmed successful insertion of each sgRNA into the corresponding vector position, verifying the successful construction of TERT gene sgRNA expression vectors (Figure 2D).

#### 3.1.3. Transfection of TERT Single-Allele Knockout Vectors and Validation of sgRNA Efficiency

**Figure 3:**
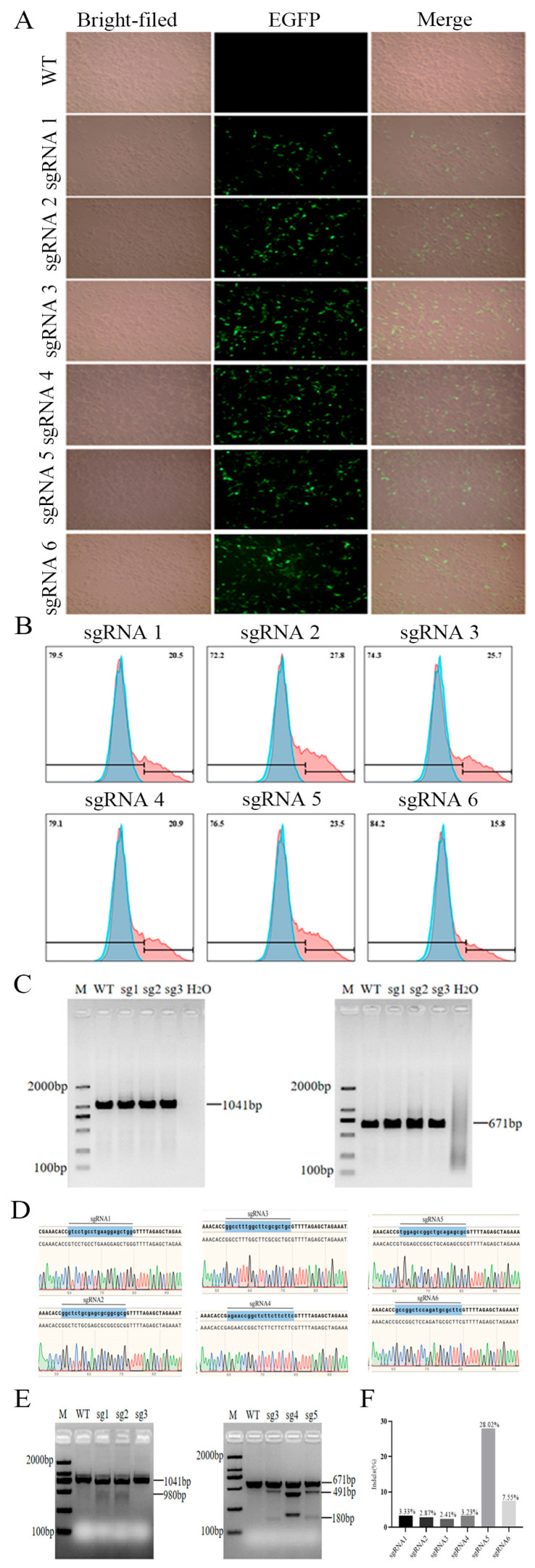
Transfection of TERT single-allele knockout vectors and validation of sgRNA efficiency. (**A**) GFP expression following 48-h transfection of PIECs with sgRNAs 1–6. Green signals indicate EGFP fluorescence. (**B**) Flow cytometry analysis and positive cell rate of PIECs transfected with sgRNAs 1–6 for 48 h. Blue and red shaded regions represent different flow cytometry populations/gating signals. (**C**) Electrophoresis analysis of PCR amplification products from EGFP-positive cells. (**D**) Sanger sequencing results of EGFP-positive cells. (**E**) T7ENI restriction analysis of EGFP-positive cell PCR products. Different peak colors represent different nucleotide signals in the Sanger sequencing chromatograms. (**F**) Statistical analysis of T7ENI cleavage efficiency for sgRNAs 1–6. Data are presented as mean ± SD from three independent biological experiments (n = 3).

The TERT gene sgRNA expression vector px458 was transfected into PIECs via liposomes. At 48 h post-transfection, a distinct GFP fluorescence signal was observed in the cells compared to untransfected wild-type controls, indicating successful cellular uptake and normal expression of the TERT sgRNA expression vector (Figure 3A). Subsequently, EGFP-positive cell populations were isolated via flow cytometry sorting, revealing transfection efficiencies of 20.5%, 27.8%, 25.1%, 20.9%, 23.5%, and 15.8% for sgRNAs 1–6, respectively (Figure 3B). PCR amplification of genomic DNA extracted from sorted cells yielded specific bands matching the anticipated sizes (Figure 3C). Following purification of PCR products, Sanger sequencing revealed distinct peak patterns with noticeable off-peak signals across all six sgRNA transfection groups, indicating targeted activity at the intended sites (Figure 3D). Further validation using T7ENI restriction enzyme digestion confirmed cleavage efficiency: WT PCR products (1041 bp/671 bp) remained intact, whereas cells transfected with sgRNAs exhibited three characteristic bands at the corresponding positions (Figure 3E). This confirmed Cas9 nuclease-mediated specific cleavage guided by sgRNAs, with cleavage efficiencies of 3.33%, 2.87%, 2.41%, 3.23%, 28.02%, and 7.55% for sgRNAs 1–6, respectively (Figure 3F). Although the cleavage efficiency of sgRNA 1 was lower than that of sgRNA 5, sgRNA 1 was the most efficient sgRNA among those targeting exon 2, while sgRNA 5 was the most efficient among those targeting exon 3. Therefore, both were selected for subsequent experiments.

#### 3.1.4. Formation of TERT Knockout PIEC Clones and Genotype Identification

**Figure 4:**
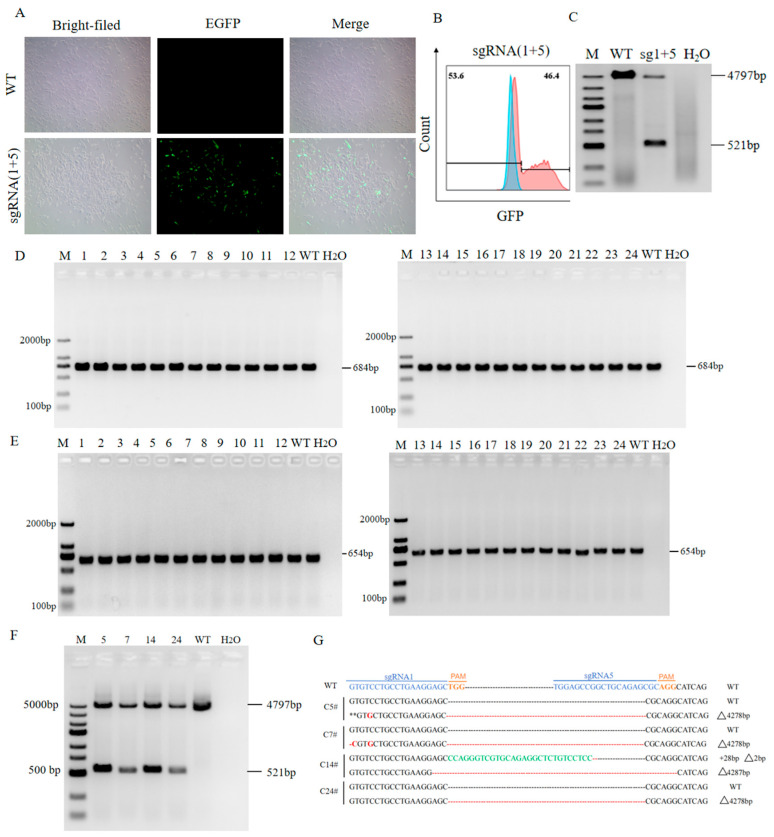
Formation and genotypic identification of TERT knockout PIEC clonal clones. (**A**) GFP fluorescence image at 48 h post-co-transfection of TERT sgRNA1 + sgRNA5. (**B**) Flow cytometry sorting results and positive rate at 48 h post-co-transfection of TERT sgRNA1 + sgRNA5. (**C**) PCR detection results for GFP-positive cells at 48 h post-co-transfection of TERT sgRNA1 + sgRNA5. (**D**) PCR amplification results for the TERT sgRNA1 target region in clones 1–24. (**E**) PCR amplification results for the TERT sgRNA5 target region in clones 1–24. (**F**) PCR amplification results for the co-targeted region of TERT sgRNA1 + sgRNA5 in clones 5, 7, 14, and 24. (**G**) Genotype sequencing results for cloning sites 5, 7, 14, and 24 (red letters denote base substitutions; red lines indicate base deletions; green letters represent base insertions; blue labels indicate sgRNA target sequences; orange labels indicate PAM sequences; red letters denote base substitutions; red dashed lines indicate deleted sequences; green letters denote inserted bases; black letters/black line segments indicate sequences identical to the WT/reference sequence; Δ indicates deletion size; “#” indicates the clone identification number; “**” indicates that the corresponding site was not covered by Sanger sequencing.

The TERT gene sgRNA1 + sgRNA5, validated for high cleavage efficiency, was co-transfected into PIECs using liposomes. At 48 h post-transfection, distinct GFP fluorescence signals were observed in cells, whereas no fluorescence was detected in untransfected WT cells, confirming successful delivery of the TERT gene sgRNA expression vector into cells (Figure 4A). Subsequently, GFP-positive cell populations were isolated via flow cytometry sorting, revealing a transfection efficiency of 46.4% (Figure 4B). During flow cytometry sorting, a portion of GFP-positive cells were isolated for monoclonal culture, while the remaining cells underwent DNA extraction for PCR validation. Results demonstrated that, compared with WT cells, those co-transfected with sgRNA 1 + sgRNA 5 amplified two specific bands (Figure 4C) matching the anticipated size. This indicates potential simultaneous cleavage at the target site by both sgRNAs, permitting progression to monoclonal screening. After culturing single-cell-derived clones for 10–14 days, a total of 24 clones were obtained. DNA extracted from these clones underwent PCR amplification, TA cloning, and sequencing analysis for genotype identification. Results showed amplification of a 684 bp target band in the TERT sgRNA 1 target region (Figure 4D) and a 654 bp target band in the TERT sgRNA 5 target region (Figure 4E), confirming no biallelic long-fragment deletion. Further PCR amplification of a long TERT gene fragment (4797 bp) revealed successful amplification of two bands in clones 5, 7, 14, and 24 (Figure 4F), confirming monoallelic long-segment deletion in these four clones. Subsequent TA cloning and sequencing analysis of these four clones revealed that clones 5, 7, and 24 exhibited a long-fragment knockout of approximately 4278 bp on one chromosome. Clone 14 demonstrated a 28-bp insertion and a 2-bp deletion on one chromosome, while the other chromosome exhibited a long-fragment knockout of approximately 4278 bp (Figure 4G). This demonstrates the successful generation of PIEC clones with TERT monoallelic knockout.

### 3.2. Construction of a TERT-Overexpressing PIEC Line

**Figure 5:**
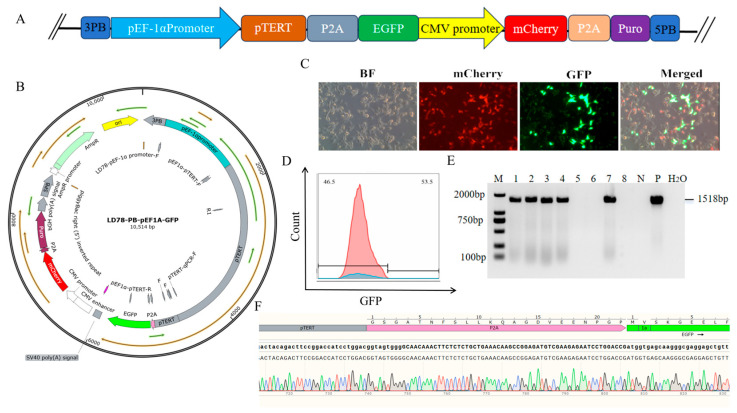
Construction of PIEC lines overexpressing the TERT gene. (**A**) Schematic representation of the PiggyBac-mediated stable overexpression strategy for the TERT gene. (**B**) Plasmid map of the TERT gene PiggyBac overexpression vector. Arrows indicate the orientation of the corresponding genetic elements, and different colors represent different functional regions/components of the construct or vector. (**C**) GFP fluorescent protein expression at 48 h post-co-transfection of cells with the TERT gene PiggyBac overexpression vector and transposase. Red fluorescence indicates mCherry expression, green fluorescence indicates GFP expression, and the merged image shows co-localization of both signals. (**D**) Flow cytometric sorting of GFP and mCherry double-positive cells. (**E**) PCR amplification results from positive clonal points. (**F**) Sanger sequencing validation of positive clonal points.

To achieve stable integration and expression of the TERT gene, this study employed the PiggyBac transposon system. This system possesses highly efficient and stable genomic integration capabilities, with its “cut-and-paste” transposition mechanism leaving virtually no molecular footprint, thereby effectively avoiding gene interference caused by residual vector sequences. Consequently, we selected the PiggyBac system to mediate stable expression of the TERT gene. First, the TERT gene was cloned into the PiggyBac transposon vector and tandemly constructed with selection markers EGFP, mCherry, and the puromycin resistance gene (PuroR) to form an expression framework (Figure 5A,B). Subsequently, this recombinant vector was co-transfected with the PiggyBac transposase expression vector into PIECs. The transposase recognised the inverted repeat sequence (IR/DR) at the ends of the transposon, excised the transposon element containing the TERT expression cassette from the plasmid, and integrated it into TTAA sites in the host genome, which are specific four-base target sequences preferentially recognised by the PiggyBac transposon system. Prominent GFP fluorescence signals were observed 48 h post-transfection (Figure 5C), confirming successful delivery of the TERT PiggyBac expression vector into PIECs. Subsequently, resistance selection was performed by adding puromycin to the cell culture medium at a final concentration of 2 μg/mL. The surviving cells were further expanded and cultured. Once the cell culture reached a stable state, GFP and mCherry double-positive cells were sorted by flow cytometry as monoclonal cultures. Flow cytometric analysis revealed a fluorescence positivity rate of 53.5% in transfected cells (Figure 5D). After 10–14 days of monoclonal culture, eight clonal points were randomly selected for molecular characterisation. Primers were designed targeting the specific region pTERT-P2A-EGFP within the integration fragment. Genomic DNA extracted from clonal point cells underwent PCR analysis. Results demonstrated successful amplification of the target 1518 bp band in clonal points 1, 2, 3, 4, and 7 (Figure 5E). Sanger sequencing revealed clear, peak-free sequencing profiles for these five clonal points, with sequences fully matching the plasmid template. This confirms successful integration of the TERT overexpression fragment into the host genome, establishing the TERT-overexpressing PIEC line (Figure 5F).

### 3.3. Validation of Senescence-Related Characteristics in TERT Monoallelic Knockout and TERT-Overexpressing PIEC Lines

**Figure 6:**
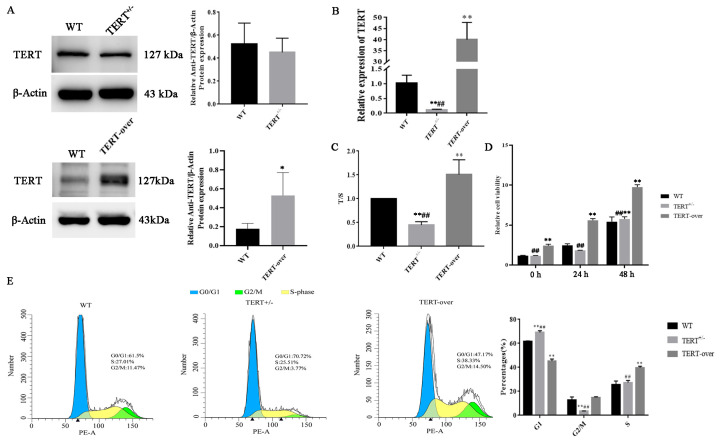
Validation of senescence-related characteristics in TERT monoallelic knockout and TERT-overexpressing PIEC lines. (**A**) Western blot analysis of TERT protein expression. WT vs. TERT+/− and WT vs. TERT-over are shown as two independent blot panels, each with its own WT control. (**B**) TERT mRNA levels in TERT+/− and TERT-over cell lines. (**C**) Telomere length determined by qPCR in each cell line, expressed as telomere amplification product (T)/single copy gene (S). (**D**) Changes in relative cell viability over 0–48 h in each cell line. (**E**) Cell cycle distribution in each cell line. Data are presented as mean ± SD from three independent biological experiments (n = 3). Triangle symbols indicate the marked peak/position of the corresponding signal. * *p* < 0.05 and ** *p* < 0.01 versus WT; ## *p* < 0.01 versus TERT-over.

To validate the efficacy of the PIEC senescent cell model constructed via monoallelic TERT gene knockout, the resulting cell lines were assessed at the protein, transcriptional, and functional levels. A TERT-overexpressing PIEC line (TERT-over) served as a comparative group for functional validation. Western blot analysis revealed markedly reduced TERT protein levels in the TERT monoallelic knockout cell line (TERT+/−) compared to the unedited wild-type (WT) control, whereas TERT protein levels were significantly elevated in the TERT-over cell line relative to WT (Figure 6A). Because the WT–TERT+/− and WT–TERT-over comparisons were analyzed in separate Western blot experiments, the WT bands shown in the two panels are not identical. Analysis of TERT mRNA levels corroborated the protein findings: TERT mRNA levels were markedly lower in TERT+/− cells compared to both WT and TERT-over cells, whereas TERT-over cells exhibited significantly elevated TERT mRNA levels relative to WT (Figure 6B). Telomere length analysis revealed that TERT monoallelic deletion resulted in significantly shorter telomeres in TERT+/− cells compared to both WT and TERT-over cells, indicating that monoallelic deletion of TERT impaired telomere maintenance and likely reduced telomerase activity (TA). Conversely, TERT-over cells exhibited significantly longer telomeres than WT cells (Figure 6C), suggesting that TERT overexpression enhanced telomere maintenance and likely increased telomerase activity (TA). Functionally, cell proliferation assays demonstrated that TERT+/− cell lines exhibited a reduced proliferation rate compared to both WT and TERT-over cell lines over 0–48 h, whereas TERT-over cells displayed markedly accelerated proliferation (Figure 6D). Cell cycle analysis further revealed that the proportion of G1-phase cells in TERT+/− cell lines was significantly increased compared to both WT and TERT-overexpressing cell lines, while the G2/M phase proportion was significantly decreased. This indicates that TERT monoallelic deletion causes cell-cycle arrest in the G1 phase. In TERT-over cell lines, the proportion of G1-phase cells was significantly lower than in WT cells, and the S-phase proportion was significantly elevated, suggesting that the enhanced cell cycle progression may be an indirect consequence of increased telomerase activity (TA) and improved telomere maintenance, including longer and better-protected telomeres, rather than a direct effect of elevated TERT expression alone (Figure 6E).

**Figure 7:**
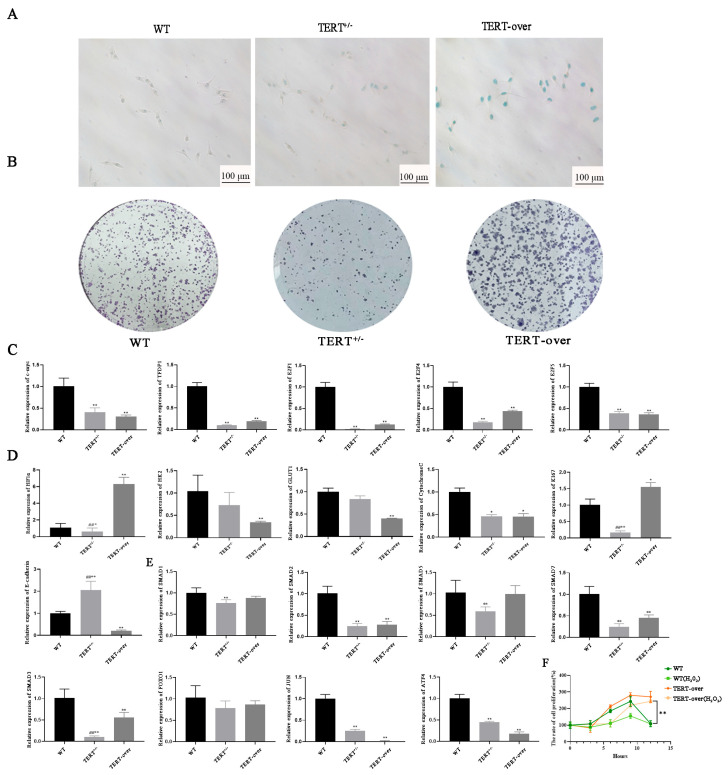
Comparative analysis of senescence-related phenotypes and oxidative stress resistance in TERT monoallelic knockout and TERT-overexpressing PIEC lines. (**A**) SA-β-gal staining results at 4 h post-culture for each cell line. (**B**) Clonogenic capacity assay results for each cell line. (**C**) mRNA levels of cell proliferation and cell cycle-related genes in each cell line. (**D**) mRNA levels of cell metabolism and energy pathway-related genes in each cell line. (**E**) mRNA levels of signal transduction and stress response-related genes in each cell line. (**F**) Cell viability of WT and TERT-over PIECs treated with 200 μmol/L H_2_O_2_ for 0, 3, 6, and 9 h, as determined by CCK-8 assay. Data are presented as mean ± SD from three independent biological experiments (n = 3). * *p* < 0.05 and ** *p* < 0.01 versus WT; ## *p* < 0.01 versus TERT-over.

SA-β-gal staining revealed markedly increased positive staining in TERT+/− cell lines compared to both WT and TERT-over lines, exhibiting a classic blue senescence phenotype, while TERT-over cells displayed the lowest positive staining proportion (Figure 7A). Clonogenic assays revealed markedly reduced clonogenic capacity in TERT+/− cells compared to both WT and TERT-over lines, whereas TERT-over cells exhibited significantly enhanced clonogenicity relative to both WT and TERT+/− cells, consistent with improved proliferative capacity that may be indirectly related to increased telomerase activity and improved telomere maintenance (Figure 7B). Furthermore, TERT+/− cells showed significant downregulation of proliferation- and cycle-regulating genes, including c-Myc, E2F family members (E2F1, E2F4, E2F5, TFDP1) (Figure 7C), and KI67 were significantly downregulated in TERT+/− cell lines compared to WT, while the adhesion molecule E-cadherin was upregulated (Figure 7D). This confirms that TERT monoallelic deletion induces G1 phase arrest and inhibits cell proliferation. Metabolic genes HIF1α, HK2, and GLUT1 were downregulated in TERT+/− cells (Figure 7D), suggesting impaired energy metabolism and altered mitochondrial-related homeostasis following TERT monoallelic deletion. In addition, signalling pathway-related genes were also altered in TERT+/− cells (Figure 7E). Several members of the SMAD family, including SMAD1, SMAD2, SMAD3, SMAD7, and SMAD9, were downregulated to varying degrees, whereas FOXO1 showed no significant change. These results suggest that TERT monoallelic deletion is associated with altered signalling-related transcription. Thus, TERT monoallelic knockout cells exhibit typical molecular hallmarks of senescence, including cell-cycle arrest, telomere shortening, reduced metabolic activity, and impaired proliferation capacity, confirming the successful establishment of this PIEC senescence model. However, in TERT-over cells, apart from the significant upregulation of HIF1α and KI67 mRNA levels compared with WT, and the significant downregulation of E-cadherin relative to both WT and TERT+/− cells, most other genes related to proliferation, metabolism, and signalling pathways showed no significant differences. To further validate the anti-ageing function of TERT-over cells, WT and TERT-over PIECs were treated with 200 μmol/L H_2_O_2_, and cell viability was assessed by CCK-8 assay at 0, 3, 6, and 9 h after treatment. Results showed that under the same treatment conditions, TERT-over cells maintained higher viability than WT cells throughout the observation period. In particular, after 9 h of H_2_O_2_ treatment, the viability of the TERT-over (H_2_O_2_) group was significantly higher than that of both the WT and WT (H_2_O_2_) groups (Figure 7F). These results suggest that TERT overexpression improves cellular resistance to oxidative stress under the experimental conditions used.

## 4. Discussion

In this study, we achieved TERT monoallelic knockout in porcine iliac endothelial cells (PIEC) using CRISPR/Cas9 technology, obtaining a cell model with telomere-dependent senescence characteristics. Simultaneously, we constructed a TERT-overexpressing PIEC line using the PiggyBac transposon system as a cell model for functional validation of the TERT gene. To our knowledge, there are currently no reports on the establishment of a senescence model in pigs using TERT monoallelic knockout strategies. This study holds significant exploratory value in the construction of large animal senescence models. Compared to commonly used experimental animals such as mice, pigs are more similar to humans in terms of organ structure, metabolic characteristics, immune system, and lifespan, and their telomere length and telomerase activity patterns are also highly similar to those of humans, thus better reflecting the physiological characteristics of telomere-related senescence processes.

The aging process is regulated by multiple organs, the immune system, and metabolic networks [17]. Previous studies using gene-edited animal models have shown that different degrees of gene disruption can result in markedly different phenotypes. For example, ATP13A2+/− mice display milder progressive changes, whereas biallelic ATP13A2 knockout mice exhibit more severe pathological phenotypes [18]. Likewise, TERT-deficient models have shown that partial loss of TERT function can reproduce progressive telomere-related changes without the severe defects seen in complete knockout models [19]. Based on this rationale, we used monoallelic TERT knockout to model gradual telomere dysfunction-associated senescence. In this study, by designing targets in the exon 2 and exon 3 regions of the TERT gene, we achieved monoallelic knockout of the TERT gene, resulting in impaired telomerase activity (TA) due to partial loss of TERT function. This led to cells exhibiting significant telomere-dependent aging characteristics, such as decreased TERT protein and mRNA levels, shortened telomere length, an increased proportion of G1 phase cells, an increased SA-β-Gal-positive cell ratio, reduced colony formation, and downregulation of proliferation-related genes, including c-Myc, E2F1, E2F4, E2F5, TFDP1, and Ki-67. These findings collectively demonstrate pronounced telomere-dependent senescence characteristics.

Furthermore, when telomere dysfunction occurs, mitochondrial homeostasis and energy metabolism are also affected [20]. In our TERT+/− cells, many of the observed phenotypes are likely secondary to reduced telomerase activity and the resulting telomere shortening, rather than representing isolated downstream effects of TERT deficiency itself. The shortened telomeres observed in these cells suggest compromised telomere capping and telomere dysfunction, which may underlie the senescence-associated phenotypes, including G1 arrest, reduced proliferation, altered metabolic status, and impaired stress-response pathways. This interpretation is in line with previous studies demonstrating that critically short or dysfunctional telomeres can drive cellular senescence and metabolic compromise. In this study, the expression of cytochrome C was downregulated in the TERT+/− cell line, accompanied by downregulation of the glycolysis-related genes HIF1α, HK2, and GLUT1. These findings suggest that monoallelic TERT knockout leads to telomere dysfunction, which may in turn contribute to mitochondrial damage and reduced metabolic activity. This is consistent with the results observed by Bao et al. [21] in diabetic nephropathy models, where TERT downregulation leads to a decrease in mitochondrial membrane potential, impaired oxidative phosphorylation activity, and reduced ATP production. That is, TERT deficiency can cause mitochondrial dysfunction and disrupt energy metabolism homeostasis. This study also found that signaling and stress response genes, such as the SMAD family, FOXO1, and ATF4, were downregulated in the TERT+/− cell line. Previous studies have shown that TERT downregulation can affect FOXO1 transcriptional regulation and the ATF4 stress pathway [22], and FOXO1 interacts with the telomere/telomerase system [23]. This suggests that TERT gene functional defects may accelerate cellular aging by inhibiting anti-stress and signaling pathways such as FOXO1-ATF4.

In this study, TERT-overexpressing cells exhibited significantly elevated TERT protein and mRNA levels, extended telomeres, accelerated cell proliferation rate, decreased G1 phase proportion, increased S phase proportion, reduced SA-β-Gal-positive proportion, and enhanced colony formation. These findings indicate that upregulation of TERT expression promotes cell cycle progression and enhances telomerase activity, exerting an anti-aging effect. The construction of TERT gene-overexpressing cell lines provides a basis for verifying the function of the TERT gene. Consistent with previous reports showing that hTERT overexpression or TERT activation can promote telomere maintenance, extend cellular lifespan, and delay senescence [7,24], TERT overexpression in our study did not significantly reverse most of the transcriptional changes observed in TERT+/− cells in genes related to proliferation, metabolism, and signaling pathways. Specifically, among the genes examined, only HIF1α and KI67 mRNA levels were increased, whereas E-cadherin was decreased. This suggests that TERT overexpression does not primarily exert its anti-aging effect through the classical c-Myc/E2F or SMAD-FOXO pathways, but may rely on non-classical mechanisms. Previous studies have shown that TERT, besides maintaining telomere length, can also localize within mitochondria to participate in oxidative stress regulation and energy metabolism maintenance [25]. The TERT gene can reduce ROS levels, minimize mitochondrial DNA damage, and stabilize membrane potential and metabolic activity [26]. In this study, TERT-overexpressing cells exhibited higher survival rates and proliferative capacity under oxidative stress conditions, suggesting improved cellular resistance to oxidative stress and enhanced cytoprotective capacity under the experimental conditions used. However, we did not directly measure antioxidant capacity or ROS-scavenging activity in this study. Therefore, these findings should not be interpreted as direct evidence of an intrinsic antioxidant function of TERT. Rather, they are consistent with previously reported non-canonical roles of TERT in mitochondrial regulation and cellular responses to oxidative stress. Additionally, TERT can interact with transcription factors such as HIF1α, NF-κB, and β-catenin to regulate cell survival and stress adaptation capabilities [27]. The upregulation of HIF1α in TERT-overexpressing cells suggests that TERT may enhance cell tolerance to oxidative and hypoxic environments, thereby activating anti-apoptotic and anti-aging responses. The increase in KI67 also indicates that TERT promotes cell proliferative potential, while the downregulation of E-cadherin suggests that TERT mediates weakened intercellular adhesion and contact inhibition, facilitating cells to enter a proliferative state.

An ideal animal model for ageing research should be capable of reproducing key features of the ageing process and exhibiting human-like metabolic and immune responses [28]. In vitro models can reveal cellular and molecular changes under controlled conditions, but lack tissue complexity and systemic metabolic environments [29,30,31]. In contrast, animal ageing models offer a more comprehensive representation of the physiological ageing process. Compared with rodents, pigs more closely resemble humans in physiological structure, metabolic characteristics, lifespan, and telomere biology [32], making them a valuable large-animal model for ageing research and anti-ageing intervention studies. This study achieved TERT monoallelic knockout and overexpression in PIECs, establishing a technical framework for constructing porcine ageing models and validating TERT gene function in cellular systems. Future applications of this technical framework in porcine fibroblasts, combined with somatic cell nuclear transfer (SCNT), may enable the generation of TERT-edited pigs. The specific significance of our model is that it provides a porcine, telomere-dysfunction-based platform for studying senescence in a species with telomere biology more similar to that of humans, and it may be particularly useful for investigating vascular endothelial ageing and related metabolic dysfunction in a large-animal setting. A limitation of the present study is that we did not directly evaluate the effect of TERT monoallelic knockout or TERT overexpression on the replicative lifespan of PIECs by long-term serial passaging. Although our data demonstrate clear differences in telomere length, proliferation, cell-cycle progression, senescence-associated markers, and resistance to oxidative stress, these findings do not by themselves fully define replicative lifespan. Direct assessment of cumulative population doublings and long-term passage capacity will therefore be necessary in future studies to determine how TERT monoallelic knockout and overexpression affect the replicative lifespan of porcine endothelial cells.

## 5. Conclusions

In summary, we successfully generated TERT monoallelic knockout and TERT-overexpressing porcine iliac artery endothelial cell lines. TERT monoallelic knockout induced marked telomere shortening, mitochondrial dysfunction, cell-cycle arrest, and suppression of stress-related pathways, indicating that loss of a single TERT allele is sufficient to trigger senescence-associated cellular changes and to mimic key features of progressive cellular aging. In contrast, TERT-overexpressing cells exhibited increased telomere length and enhanced proliferative capacity. Although the overall mRNA levels of many genes related to proliferation, metabolism, and signaling pathways were not broadly increased, the antioxidant capacity of these cells was significantly improved. These findings suggest that TERT overexpression confers a partial anti-aging phenotype, possibly through the regulation of ROS metabolism and cellular energy homeostasis. Although the present study mainly focused on the construction of porcine cell lines and the characterization of their initial cellular phenotypes, its significance lies in establishing a comparative porcine cell platform for future mechanistic studies of TERT function, telomere dysfunction, and cellular senescence in a large-animal context. A limitation of the present study is that the findings are currently restricted to the cellular level, without direct long-term evaluation of replicative lifespan or validation in gene-edited pigs. Taken together, the technical system established in this study may provide a practical basis for further gene editing in porcine fibroblasts and for the future generation of TERT gene-edited pig models using somatic cell nuclear transfer (SCNT). The construction of TERT-edited pig models will be an important next step to extend the present findings from the cellular level to the whole-animal level and to further evaluate the physiological significance of TERT in ageing-related processes. Such models may be useful for investigating the mechanisms of multi-organ aging, cardiovascular dysfunction, and metabolic imbalance associated with telomere dysfunction, and may also provide valuable support for future translational studies.

## Data Availability

The data that support the findings of this study are available on request from author 1 or the corresponding author.

## Abbreviations

The following abbreviations are used in this manuscript:

TERT	telomerase reverse transcriptase
TERC	telomerase RNA component
TA	telomerase activity
PIEC	porcine iliac artery endothelial cells
SCNT	somatic cell nuclear transfer
SNP	single-nucleotide polymorphism
qPCR	quantitative polymerase chain reaction
WT	wild type
SA-β-gal	senescence-associated beta-galactosidase
EGFP	enhanced green fluorescent protein
PBS	phosphate-buffered saline
PBST	phosphate-buffered saline with Tween-20
BSA	bovine serum albumin
RIPA	radioimmunoprecipitation assay
PI	propidium iodide
FSC	forward scatter
SSC	side scatter
CCK-8	Cell Counting Kit-8
PBL	peripheral blood lymphocytes
UCB	umbilical cord blood
PuroR	puromycin resistance gene
IR/DR	inverted repeat/direct repeat
